# Sex and Feeding Status Differently Affect Natural Reward Seeking Behavior in Olfactory Bulbectomized Rats

**DOI:** 10.3389/fnbeh.2018.00255

**Published:** 2018-10-30

**Authors:** Jana Ruda-Kucerova, Mary Tresa Zanda, Petra Amchova, Walter Fratta, Liana Fattore

**Affiliations:** ^1^Department of Pharmacology, Faculty of Medicine, Masaryk University, Brno, Czechia; ^2^Department of Biomedical Sciences, Section of Neuroscience and Clinical Pharmacology, University of Cagliari, Monserrato, Italy; ^3^Center of Excellence “Neurobiology of Addiction”, University of Cagliari, Monserrato, Italy; ^4^CNR Institute of Neuroscience-Cagliari, National Research Council, Rome, Italy

**Keywords:** self-administration, food intake, olfactory bulbectomy, depression, sex difference, reward

## Abstract

Substance abuse and depression are common psychiatric disorders with a high rate of comorbidity. Both conditions affect differently men and women and preclinical research has showed many sex differences in drug addiction and depression. The most common approach for modeling depression-addiction comorbidity is the combination of the intravenous drug self-administration and the olfactory bulbectomy (OBX) models in rats. Such a combination has revealed enhanced drug-taking and drug-seeking behaviors in OBX rats, but no study has investigated so far potential sex differences in operant responding and motivation for natural reinforcers in OBX rats. This study investigated for the first time operant self-administration of palatable food pellets in male and female OBX rats under different feeding status, i.e., *ad libitum* vs. restricted food, and schedules of reinforcement, i.e., a continuous ratio schedule fixed ratio 1 (FR1) vs. a complex (FR5_(x)_) second order schedule of reinforcement. In the FR1 experiment, OBX rats of both sexes exhibited lower operant responding and intake of palatable food pellets than sham-operated controls, with food restriction leading to increased operant responding in both OBX and SHAM groups. Female rats showed higher responding than males but this effect was abolished by the OBX lesion. Similarly, in the (FR5_(x)_) second order schedule of reinforcement both male and female OBX rats showed lower responding and food intake, with SHAM and OBX females showing higher operant responding than corresponding male groups. Overall, our findings showed that: (i) responding for food was lower in OBX than in SHAM rats under both FR1 and (FR5_(x)_) schedules of reinforcement; (ii) sex and food restriction affect operant responding for palatable food; and (iii) the suppressing effect of OBX lesion on food intake was consistently present in both sexes and represents the most robust factor in the analysis. This may represent anhedonia which is associated with depressive-like phenotype and palatable food self-administration may serve as a robust behavioral index of anhedonia in the OBX model.

## Introduction

Substance use disorder and depression are common in the general population and display a high frequency of comorbidity (Torrens and Rossi, 2015). In humans, gender differences have been consistently described in substance use disorder (Fattore et al., 2008; Fattore and Melis, 2016) and depression (Marcus et al., 2005) as well as in other forms of addictive behaviors (Fattore et al., 2014) and emotional disturbances (Seney and Sibille, 2014). In depressed substance users it is hard to both determine if one disorder (e.g., depression) may affect predisposition to the other (e.g., drug abuse) and identify the underlying mechanisms. Animal studies experimentally reproduce behavioral traits and features relevant to depression and drug intake and allow the study of the underlying mechanisms. Despite inevitable limitations, animal models are unanimously acknowledged as essential for understanding the biological factors that contribute to addiction and mood disorders and for developing new pharmacotherapies (Micale et al., 2013; Müller, 2017; Robinson, 2018). They also represent an essential step in the study of sex differences (Becker and Koob, 2016) and motivational brain system (Gonen et al., 2012) as well as in the development of tailored therapeutic strategies for male and female patients (Buoncervello et al., 2017).

Reliable and validated animal models are currently available to study addiction and depression. Yet, only relatively few preclinical studies have examined addictive behaviors using animal models of depression (Filip et al., 2013) and even less are those that performed the experiments in both male and female animals to investigate potential sex differences (Ng et al., 2017). Operant behavior to obtain palatable food strongly impacts upon the brain reward circuit (Guegan et al., 2013) and, in animals, operant responding for food reinforcers has been shown to be affected by sex (Ward and Walker, 2009). Food intake is strictly associated to emotional states by complex physiological and behavioral interactions (Ulrich-Lai et al., 2015), and eating behavior can be significantly altered in depressed subjects (Paans et al., 2018).

The olfactory bulbectomy (OBX) in rats is a widely recognized animal model that displays a high degree of neurochemical similarity to human depression, helps to identify potential antidepressant drugs and gives important insights into their possible mechanisms of action (Leonard, 1984; van Riezen and Leonard, 1990; Kelly et al., 1997; Harkin et al., 2003; Song and Leonard, 2005). A number of self-administration studies have been conducted in OBX rats to verify whether depressive-like animals displayed altered voluntary intake of drugs of abuse. Findings have shown that, when compared to corresponding sham-operated animals, OBX rats exhibit a higher intake of amphetamine (Holmes et al., 2002), nicotine (Vieyra-Reyes et al., 2008), methamphetamine (Kucerova et al., 2012), synthetic cannabinoid (Amchova et al., 2014) and ketamine (Babinska and Ruda-Kucerova, 2017), but not of cocaine (Frankowska et al., 2014), suggesting that OBX rats may have a different sensitivity to the pharmacological effects of drugs of abuse. Notably, neural circuits regulating drug-induced reward extensively overlap with those regulating natural reward and reinforcement, including food (Blum et al., 2011). Interestingly, bulbectomized rats were found to display pronounced deficits in brain reward functions (Slattery et al., 2007; Grecksch and Becker, 2015) associated to a dysfunctional dopaminergic signaling (Amchova et al., 2014; Jastrzębska et al., 2015; Ruda-Kucerova et al., 2015a), which suggests that the reward mechanisms may be altered in this animal model as a common phenomenon associated with depression. The hypothesis of a brain hypodopaminergic function induced by bilateral OBX is strengthened by the finding that sucrose preference is reduced, i.e., anhedonia (Romeas et al., 2009; Sato et al., 2010) and the expression of cocaine-induced place preference is disrupted in OBX rats (Calcagnetti et al., 1996). OBX was also shown to modulate feeding pattern in rats (Meguid et al., 1993), with functional adaptations of food intake regulatory mechanism occurring over time after olfactory bulbs ablation (Meguid et al., 1997). Yet, whether operant responding for palatable food and underlying motivation is altered in OBX rats remains an open question. Equally unexplored are the potential factors that may affect self-administration of palatable food in OBX rats, such as the diet regimen and the amount of effort required to obtain a single pellet of palatable food.

This study was designed to: (i) assess if alterations in the drug self-administration reported in OBX rats extend to operant responding and motivation for natural reinforcers, i.e., palatable food; (ii) verify whether a depression-like state may affect operant behavior differently in male and female rats; and (iii) determine the potential modulating role of the feeding status (i.e., restricted vs. *ad libitum*) and the reinforcement schedule complexity fixed ratio 1 (FR1) vs. (FR5_(x)_) second order schedule) in operant responding for food in OBX rats.

## Materials and Methods

### Animals

Thirty-six male and 36 female Lister-Hooded rats (weight range of 250–300 g at the beginning of the experiment) were purchased from Harlan-Nossan (Italy) and housed four per cage at the Animal Facility of the Department of Biomedical Sciences, University of Cagliari, Italy. Males and females were housed in different rooms. Environmental conditions during the whole study were constant: relative humidity 50%–60%, room temperature 23°C ± 1°C, inverted 12-h light-dark cycle (6 a.m. to 6 p.m. darkness). Food and water were available *ad libitum* unless otherwise specified below. All procedures were performed in accordance with EU Directive no. 2010/63/EU and approved by the local Animal Care Committee at the Department of Biomedical Sciences, University of Cagliari, Italy.

### Olfactory Bulbectomy Surgery

At the beginning of the experiments, both male and female rats were randomly divided into two groups (*n* = 18 per group): bulbectomized (OBX) and sham-operated (SHAM) rats. The bilateral ablation of the olfactory bulbs was performed as previously described (Amchova et al., 2014; Ruda-Kucerova et al., 2015a). Animals were anesthetized with isofluran 2%, the top of the skull was shaved and swabbed with an antiseptic solution. Then, midline frontal incision was made on the skull and the skin was retracted bilaterally. Two burr holes, 2 mm in diameter, were drilled in the frontal bone 7 and 7.5 mm anterior from the Bregma, 1.5 and 2 mm lateral to Bregma suture for rats weighing 230 ± 10 g and 260 ± 10 g, respectively. Both olfactory bulbs were removed by aspiration paying particular attention to not damage the frontal cortex. Prevention of blood loss from the ablation cavity was achieved by filling the dead space with a hemostatic sponge. The skin above the lesion was closed with suture. Finally, bacitracin plus neomycin powder was applied to prevent bacterial infection. Sham-operated rats underwent identical anesthetic and drilling procedures but their bulbs were left intact. A period of at 3 weeks was allowed for the recovery from the surgical procedure and the development of the characteristic phenotype. During this period, animals were handled daily for few minutes to eliminate aggression, which could otherwise arise (Kelly et al., 1997; Song and Leonard, 2005). At the end of the experiment, rats were euthanized by an anesthetic overdose and the brains were dissected for confirmation of the successful surgical lesion. Three animals (1 F, 2 M) were excluded from analysis due to incomplete removal of the OB, while one female rat was excluded because of damage to the prefrontal cortex.

### Food Self-Administration Protocols

Food self-administration was conducted in 12 operant chambers (29.5 × 32.5 × 23.5 cm, Med Associates, Fairfax, VT, USA) using lever-pressing as *operandum*. Each chamber was encased in a sound and light attenuating cube. In addition, chambers had a ventilation fan, and a front panel equipped with two retractable levers (each 4 cm wide) positioned 12 cm apart, 8 cm from the grid and extending 1.5 cm into the box. A white stimulus light was placed above each lever and a red house light was located on the opposite wall. All self-administration sessions were conducted at the same time daily during the dark period of the inverted light-dark cycle.

There were two experiments performed. **Experiment 1 (FR1)** aimed to determine consummatory behavior and evaluated the effect of OBX and feeding status (*ad libitum* vs. food restriction to 80% of free-feeding body weight). For this part of the study the rats were divided into following groups (*n* = 6 per group): SHAM fed *ad libitum*, SHAM restricted, OBX fed *ad libitum* and OBX restricted. **Experiment 2 (FR5_(x)_)** was designed to assess appetitive behavior. In this experiment, the number of groups was reduced due to equal minimal effect of the food restriction observed in the Experiment 1. Therefore, rats were only divided into two groups, *n* = 6 per group: SHAM and OBX. This allowed a reduction of rats used and refinement of the procedures by including only *ad libitum* fed rats.

#### Experiment 1 (FR1)

The training was conducted under a FR1 schedule of reinforcement, i.e., the animal had to press to the active lever once to obtain a single palatable food pellet (BioServ, palatable dustless rodent food pellets, F0021-Purified Casein Based Formula—45 mg). The session lasted 30 min and the house-light was on throughout the session. The length of the training was 10 consecutive days. All animals consumed the vast majority of the gained pellets of food. There were four experimental groups of male rats (*n* = 6) and four groups of female rats (*n* = 6) divided by the OBX surgery and feeding status. Data are expressed as mean of active/inactive lever presses per group and session; gained food pellets were analyzed as a cumulative count, e.g., “day 5 cumulative intake” equals the sum of the number of food pellets self-administered on days 1–5).

#### Experiment 2 (FR5_(x)_)

A different batch of SHAM and OBX rats was used in the Experiment 2. The training was conducted under a (FR5_(x)_) second order protocol as described by Thornton-Jones et al. (2005) with slight modifications. Briefly, animals were first trained to lever press on a FR1 schedule for 7–8 days. A single press on the active lever led to the illumination of a house-light and delivery of a single food pellet after 4 s. Inactive lever presses were not rewarded but the house-light was on, indicating that the FR requirement has been fulfilled. The house-light remained on for 4 s prior to food pellets delivery and 4 s after delivery to enhance the cue effect of the light. Once animals reached more than 100 active lever presses on FR1, they were transferred to an FR5 schedule. The protocol was analogous to FR1 but rats were required to press five times the active lever to get a single food pellet and lasted 4 days. After completion rats were transferred to a FR5(2) schedule and were required to obtain two consecutive house-light presentations in order to receive two food pellets (4 days) followed by FR5(3) schedule in a similar manner, i.e., three house-light presentations to receive three food pellets (4 days) and FR5(4) schedule (4 days). Finally, the animals underwent the final training schedule FR5(5) for 7 days. There were two experimental groups of male rats (SHAM and OBX, *n* = 6) and two groups of female rats (SHAM and OBX, *n* = 6). Data are expressed as mean of active/inactive lever presses and number of food pellets per group and session.

### Statistical Data Analysis

Primary data were summarized using arithmetic mean and standard error of the mean (SEM) estimate. The analyses were calculated using Statistica 12 (StatSoft, Inc. Tulsa, OK, USA). A value *p* < 0.05 was recognized as boundary of statistical significance in all applied tests.

#### Experiment 1

Food self-administration data were analyzed by ANOVA for repeated measures (RM) with factors: OBX model, feeding status, repeated variable: day; followed by Bonferroni *post hoc* test for analysis of significant interactions of factors. Sex differences in the food self-administration were also assessed RM ANOVA (factors: OBX model, sex, repeated variable: day) followed by Bonferroni *post hoc* test for analysis of significant interactions of factors.

#### Experiment 2

Food self-administration data were analyzed by RM ANOVA with factors: OBX model, repeated variable: FR schedule; followed by Bonferroni *post hoc* test for analysis of significant interactions of factors. Sex differences were assessed by RM ANOVA (factors: OBX model, sex, repeated variable: FR schedule) followed by Bonferroni *post hoc* test for analysis of significant interactions of factors.

## Results

In line with previous study conducted on hamsters (Pieper et al., 1992) and rats (Meguid et al., 1993), bilateral OBX did not alter food intake and body weight gain, and these parameters did not differ significantly through the study between OBX and SHAM groups of the same sex.

### Experiment 1 (FR1 Schedule)

Experiment 1 assessed operant self-administration of palatable food pellets over 10 days under FR1 schedule of reinforcement in both male and female Lister Hooded rats. The data are presented as daily mean numbers of active lever presses, inactive lever presses and cumulative number of delivered food pellets.

#### Male Rats

**Figure 1:**
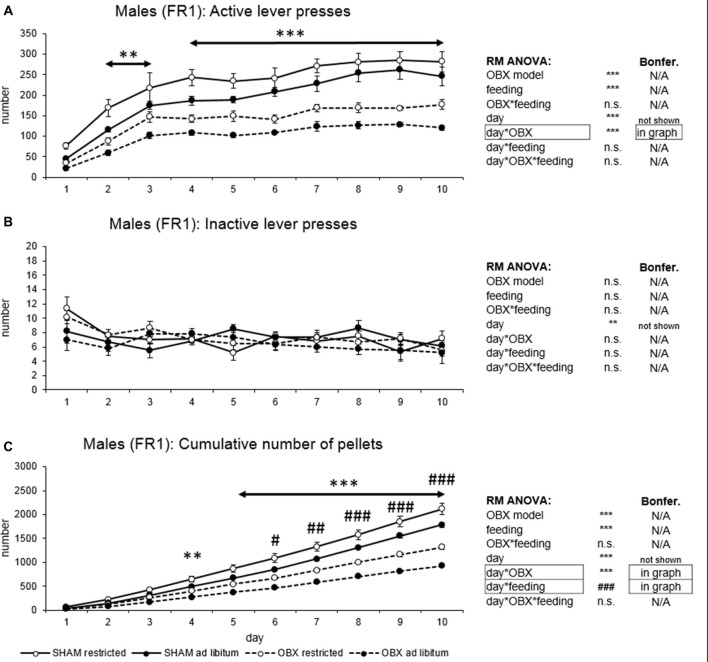
Self-administration of palatable pellets in MALE rats under fixed ratio 1 (FR1) schedule. The graphs present the mean ± standard error of the mean (SEM) of numbers of active lever presses **(A)**, inactive lever presses **(B)** and cumulative numbers of self-administered pellets **(C)** in male rats during the 10-days training. The tables on the side summarize results of repeated measures (RM) ANOVA and Bonferroni *post hoc* test for each variable (**p < 0.01, ***p < 0.001 for day*olfactory bulbectomy (OBX) interaction, ^#^*p* < 0.05, ^##^*p* < 0.01, ^###^*p* < 0.001 for day*feeding interaction).

In active lever pressing (Figure 1A) RM ANOVA revealed a highly significant effect of OBX model (*F*_(1,20)_ = 123.65, *p* < 0.001), feeding status (*F*_(1,20)_ = 22.37, *p* < 0.001), day (*F*_(9,180)_ = 49.76, *p* < 0.001) and day*OBX model interaction (*F*_(9,180)_ = 3.57, *p* < 0.001). Bonferroni *post hoc* test for day*OBX model interaction indicated significantly decreased responding in the OBX rats from day 2 onwards (day 2: *t*_(158,38)_ = 4.20, *p* = 0.009; day 3: *t*_(158,38)_ = 4.45, *p* = 0.003; day 4: *t*_(158,38)_ = 5.49, *p* < 0.001; day 5: *t*_(158,38)_ = 5.30, *p* < 0.001; day 6: *t*_(158,38)_ = 6.13, *p* < 0.001; day 7: *t*_(158,38)_ = 6.37, *p* < 0.001; day 8: *t*_(158,38)_ = 7.32, *p* < 0.001; day 9: *t*_(158,38)_ = 7.68, *p* < 0.001; day 10: *t*_(158,38)_ = 7.09, *p* < 0.001). Inactive lever pressing did not differ among the groups (Figure 1B) and RM ANOVA indicated only a significant effect of the repeated factor—day (*F*_(9,180)_ = 2.81, *p* = 0.004) which reflects the learning process during acquisition.

Cumulative number of delivered food pellets (Figure 1C) showed similar trends as active responding. RM ANOVA showed a highly significant effect of OBX model (*F*_(1,20)_ = 81.42, *p* < 0.001), feeding status (*F*_(1,20)_ = 20.99, *p* < 0.001), day (*F*_(9,180)_ = 1175.89, *p* < 0.001), day*OBX model interaction (*F*_(9,180)_ = 81.90, *p* < 0.001) and day*feeding status interaction (*F*_(9,180)_ = 14.80, *p* < 0.001). Bonferroni *post hoc* test for day*OBX model interaction indicated significantly suppressed food pellet intake in the OBX rats from day 4 onwards (day 4: *t*_(41,118)_ = 4.55, *p* = 0.009; day 5: *t*_(41,118)_ = 6.0, *p* < 0.001; day 6: *t*_(41,118)_ = 7.85, *p* < 0.001; day 7: *t*_(41,118)_ = 9.54, *p* < 0.001; day 8: *t*_(41,118)_ = 11.70, *p* < 0.001; day 9: *t*_(41,118)_ = 13.96, *p* < 0.001; day 10: *t*_(41,118)_ = 16.19, *p* < 0.001). Bonferroni *post hoc* test for day*feeding status interaction revealed higher food pellet intake in the food restricted animals from day 6 onwards (day 6: *t*_(41,12)_ = 4.30, *p* = 0.02; day 7: *t*_(41,12)_ = 4.97, *p* = 0.002; day 8: *t*_(41,12)_ = 5.63, *p* < 0.001; day 9: *t*_(41,12)_ = 6.31, *p* < 0.001; day 10: *t*_(41,12)_ = 7.12, *p* < 0.001). All variables are shown in Figure 1 together with an overview of statistical results (right).

#### Female Rats

**Figure 2:**
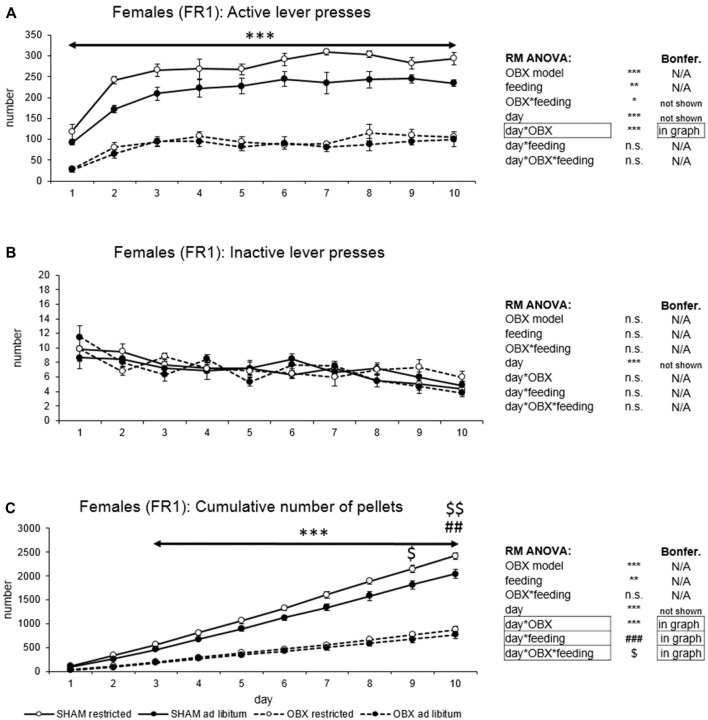
Self-administration of palatable pellets in FEMALE rats under FR1 schedule. The graphs present the mean ± SEM of numbers of active lever presses **(A)**, inactive lever presses **(B)** and cumulative numbers of self-administered pellets **(C)** in female rats during the 10-days training. The tables on the side summarize results of RM ANOVA and Bonferroni *post hoc* test for each variable (***p < 0.001 for day*OBX interaction, ^##^*p* < 0.01 for day*feeding status interaction, ^$^*p* < 0.05, ^$$^*p* < 0.01 for day*OBX feeding status interaction—SHAM restricted vs. SHAM *ad libitum*).

In active lever pressing (Figure 2A) RM ANOVA revealed a highly significant effect of OBX model (*F*_(1,20)_ = 275.57, *p* < 0.001), feeding status (*F*_(1,20)_ = 11.25, *p* = 0.003) and OBX model*feeding status interaction (*F*_(1,20)_ = 5.36, *p* = 0.031). Bonferroni *post hoc* test showed increased responding in SHAM restricted rats compared to *ad libitum* fed animals (*t*_(20,00)_ = 4.01, *p* = 0.004) while the comparison of OBX restricted and *ad libitum* fed animals renders non-significant result. Furthermore, RM ANOVA showed a significant effect of day (*F*_(9,180)_ = 34.00, *p* < 0.001) and day*OBX model interaction (*F*_(9,180)_ = 6.08, *p* < 0.001). Bonferroni *post hoc* test for day*OBX model interaction indicated significantly lower responding in the OBX rats in all 10 consecutive sessions (day 1: *t*_(109,41)_ = 5.19, *p* < 0.001; day 2: *t*_(109,41)_ = 8.94, *p* < 0.001; day 3: *t*_(109,41)_ = 9.55, *p* < 0.001; day 4: *t*_(109,41)_ = 9.62, *p* < 0.001; day 5: *t*_(109,41)_ = 10.66, *p* < 0.001; day 6: *t*_(109,41)_ = 11.93, *p* < 0.001; day 7: *t*_(109,41)_ = 12.50, *p* < 0.001; day 8: *t*_(109,41)_ = 11.46, *p* < 0.001; day 9: *t*_(109,41)_ = 10.78, *p* < 0.001; day 10: *t*_(109,41)_ = 10.74, *p* < 0.001). As in male rats, inactive lever pressing did not differ among groups (Figure 2B) and RM ANOVA indicated only a significant effect of the repeated factor—day (*F*_(9,180)_ = 47.16, *p* < 0.001).

In the analysis of cumulative number of delivered food pellets (Figure 2C) RM ANOVA showed a highly significant effect of OBX model (*F*_(1,20)_ = 249.55, *p* < 0.001), feeding status (*F*_(1,20)_ = 8.80, *p* = 0.007), day (*F*_(9,180)_ = 883.33, *p* < 0.001), day*OBX model interaction (*F*_(9,180)_ = 192.26, *p* < 0.001), day*feeding status interaction (*F*_(9,180)_ = 6.03, *p* < 0.001) and day*OBX model*feeding status interaction (*F*_(9,180)_ = 2.17, *p* = 0.026). Bonferroni *post hoc* test for day*OBX model interaction indicated significantly decreased food pellet intake in the OBX rats from day 3 onwards (day 3: *t*_(42,207)_ = 6.03, *p* < 0.001; day 4: *t*_(42,207)_ = 8.45, *p* < 0.001; day 5: *t*_(42,207)_ = 11.15, *p* < 0.001; day 6: *t*_(42,207)_ = 14.16, *p* < 0.001; day 7: *t*_(42,207)_ = 17.15, *p* < 0.001; day 8: *t*_(42,207)_ = 20.08, *p* < 0.001; day 9: *t*_(42,207)_ = 22.74, *p* < 0.001; day 10: *t*_(42,207)_ = 25.49, *p* < 0.001). Bonferroni *post hoc* test for day*feeding status interaction revealed higher food pellet intake in the food restricted animals on day 9 and 10 (day 9: *t*_(42,21)_ = 4.19, *p* = 0.026; day 10: *t*_(42,21)_ = 4.70, *p* = 0.005) and Bonferroni *post hoc* test for day*OBX model*feeding status interaction showed higher food pellet intake in the food restricted SHAM animals compared to *ad libitum* fed SHAM control on days 9–10 (day 9: *t*_(42,21)_ = 4.73, *p* = 0.020, day 10: *t*_(42,21)_ = 5.37, *p* = 0.002). There was no effect of feeding status in the OBX animals. All variables are shown in Figure 2 together with an overview of statistical results (right).

#### Evaluation of Sex Differences

**Figure 3:**
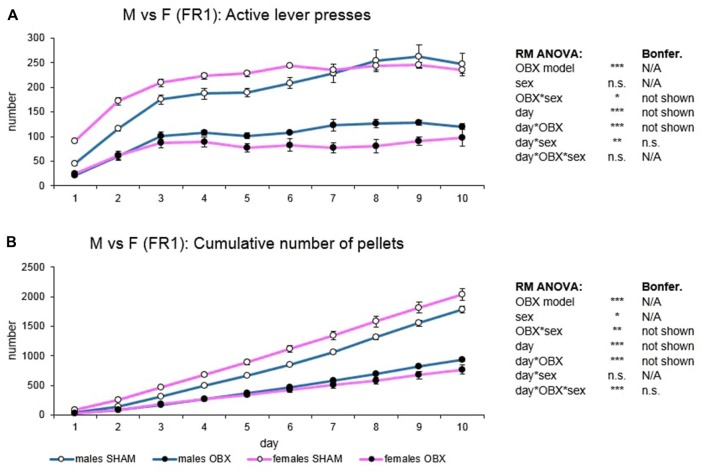
Sex differences in the self-administration of palatable pellets under FR1 schedule. The graphs present the mean ± SEM of daily numbers of active lever presses **(A)** and cumulative numbers of self-administered pellets **(B)** in *ad libitum* fed experimental groups of both sexes during the 10-days training. The tables on the side summarize results of RM ANOVA and Bonferroni *post hoc* test for each variable (*p < 0.05, **p < 0.01, ***p < 0.001). There is no sex difference in the repeated design. For the main effect of sex, see the “Results” section.

For the analysis of sex differences only the *ad libitum* fed groups were used and therefore the analyzed groups were the following: males SHAM, males OBX, females SHAM, females OBX (Figure 3). Variables included in the analysis were active lever presses and cumulative food pellets intake, responding on the inactive lever not differing among groups.

In active lever pressing (Figure 3A) RM ANOVA revealed a highly significant effect of the OBX model (*F*_(1,20)_ = 196.45, *p* < 0.001) and OBX model*sex interaction (*F*_(1,20)_ = 6.33, *p* = 0.021). Bonferroni *post hoc* test for OBX model*sex interaction indicated significantly lower responding in the male OBX rats compared to male SHAM rats (*t*_(20,00)_ = 8.13, *p* < 0.001) and female OBX rats responding less than female SHAM rats (*t*_(20,00)_ = 11.69, *p* < 0.001). Furthermore, RM ANOVA showed a significant effect of the day (*F*_(9,180)_ = 43.94, *p* < 0.001), day*OBX model (*F*_(9,180)_ = 5.90, *p* < 0.001) and day*sex interaction (*F*_(9,180)_ = 2.69, *p* = 0.006). Bonferroni *post hoc* test for day*OBX model interaction revealed lower responding in the OBX animals from day 2 onwards (day 2: *t*_(137,08)_ = 5.66, *p* < 0.001; day 3: *t*_(137,08)_ = 6.56, *p* < 0.001; day 4: *t*_(137,08)_ = 7.18, *p* < 0.001; day 5: *t*_(137,08)_ = 8.10, *p* < 0.001; day 6: *t*_(137,08)_ = 8.79, *p* < 0.001; day 7: *t*_(137,08)_ = 9.03, *p* < 0.001; day 8: *t*_(137,08)_ = 9.83, *p* < 0.001; day 9: *t*_(137,08)_ = 9.88, *p* < 0.001; day 10: *t*_(137,08)_ = 9.05, *p* < 0.001) and Bonferroni *post hoc* test for the day*sex interaction did not show any significant result.

In the cumulative number of delivered food pellets (Figure 3B) RM ANOVA showed a highly significant effect of OBX model (*F*_(1,20)_ = 202.73, *p* < 0.001), sex (*F*_(1,20)_ = 4.45, *p* = 0.048), and OBX model*sex interaction (*F*_(1,20)_ = 13.55, *p* = 0.001). Bonferroni *post hoc* test for OBX model*sex interaction indicated significantly higher food pellets intake in the female SHAM rats compared to male SHAM rats (*t*_(20,00)_ = −4.09, *p* = 0.003) but not in the OBX groups: female OBX rats compared to male OBX rats (n.s.). Furthermore, RM ANOVA detected a significant effect of day (*F*_(9,180)_ = 888.39, *p* < 0.001), day*OBX model interaction (*F*_(9,180)_ = 131.27, *p* < 0.001), and day*OBX model*sex interaction (*F*_(9,180)_ = 4.83, *p* < 0.001). Bonferroni *post hoc* test for day*OBX model interaction revealed higher responding in the SHAM animals from day 3 onwards (day 3: *t*_(52,23)_ = 4.58, *p* < 0.001; day 4: *t*_(52,23)_ = 6.78, *p* < 0.001; day 5: *t*_(52,23)_ = 9.18, *p* < 0.001; day 6: *t*_(52,23)_ = 11.70, *p* < 0.001; day 7: *t*_(52,23)_ = 14.35, *p* < 0.001; day 8: *t*_(52,23)_ = 17.41, *p* < 0.001; day 9: *t*_(52,23)_ = 20.27, *p* < 0.001; day 10: *t*_(52,23)_ = 23.01, *p* < 0.001) and Bonferroni *post hoc* test for the day*OBX model*sex interaction did not show any significant results.

### Experiment 2 (FR5_(x)_)

Experiment 2 evaluated operant self-administration of palatable food pellets under a complex FR5 schedule of reinforcement (FR5_(x)_) in both sexes of Lister Hooded rats. The factor of feeding status was eliminated and animals were all fed *ad libitum*. Data are presented as mean numbers of active lever presses, inactive lever presses and cumulative number of delivered food pellets during the particular schedule (see “Materials and Methods” section).

#### Male Rats

**Figure 4:**
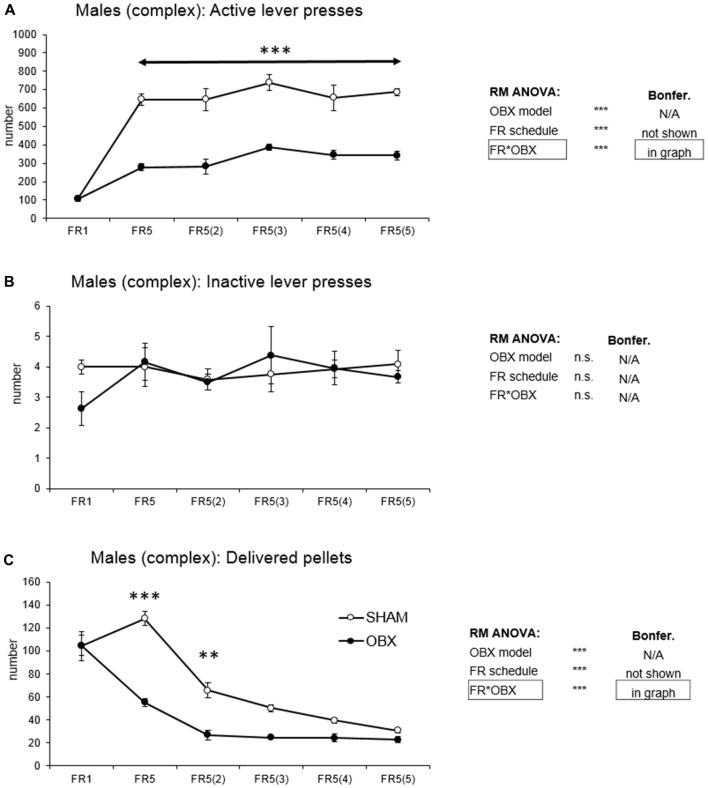
Self-administration of palatable pellets in MALE rats under complex FR5_(x)_ schedule. The graphs present the mean ± SEM of mean numbers of active lever presses **(A)**, inactive lever presses **(B)** and self-administered pellets **(C)** in *ad libitum* fed SHAM and OBX male rats during each schedule of the complex protocol. The tables on the side summarize results of RM ANOVA and Bonferroni *post hoc* test for each variable (**p < 0.01, ***p < 0.001).

In active lever pressing (Figure 4A) RM ANOVA revealed a highly significant effect of OBX model (*F*_(1,10)_ = 103.63, *p* < 0.001), schedule of reinforcement (*F*_(5,50)_ = 42.49, *p* < 0.001) and schedule of reinforcement*OBX model interaction (*F*_(5,50)_ = 7.89, *p* < 0.001). Bonferroni *post hoc* test for schedule of reinforcement*OBX model interaction indicated significantly decreased active responding in the OBX rats on all FR5_(x)_ protocols (FR5: *t*_(55,716)_ = −6.75, *p* < 0.001; FR5(2): *t*_(55,716)_ = −6.67, *p* < 0.001; FR5(3): *t*_(55,716)_ = −6.46, *p* < 0.001; FR5(4): *t*_(55,716)_ = −5.56, *p* < 0.001; FR5(5): *t*_(55,716)_ = −6.30, *p* < 0.001). Inactive lever pressing did not differ among the groups.

Mean number of delivered food pellets (Figure 4C) showed a highly significant effect of OBX model (*F*_(1,10)_ = 50.18, *p* < 0.001), schedule of reinforcement (*F*_(5,50)_ = 58.89, *p* < 0.001) and schedule of reinforcement*OBX model interaction (*F*_(5,50)_ = 9.36, *p* < 0.001). Bonferroni *post hoc* test for schedule of reinforcement*OBX model interaction indicated significantly decreased food pellets intake in the OBX rats in the FR5 (*t*_(59,838)_ = −8.33, *p* < 0.001) and FR5(2) (*t*_(59,838)_ = −4.45, *p* = 0.002) protocols. All variables are shown in Figure 4 together with an overview of statistical results (right).

#### Female Rats

**Figure 5:**
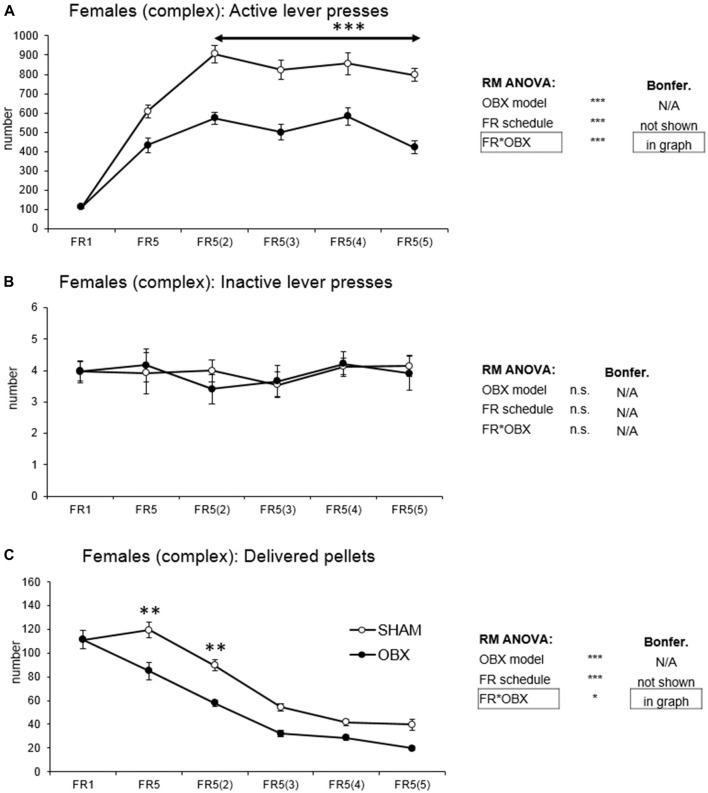
Self-administration of palatable pellets in FEMALE rats under complex FR5_(x)_ schedule. The graphs present the mean ± SEM of mean numbers of active lever presses **(A)**, inactive lever presses **(B)** and self-administered pellets **(C)** in *ad libitum* fed SHAM and OBX female rats during each schedule of the complex protocol. The tables on the side summarize results of RM ANOVA and Bonferroni *post hoc* test for each variable (*p < 0.05, **p < 0.01, ***p < 0.001).

In active lever pressing (Figure 5A) RM ANOVA revealed a highly significant effect of OBX model (*F*_(1,10)_ = 71.37, *p* < 0.001), schedule of reinforcement (*F*_(5,50)_ = 69.78, *p* < 0.001) and schedule of reinforcement*OBX model interaction (*F*_(5,50)_ = 6.15, *p* < 0.001). Bonferroni *post hoc* test for schedule of reinforcement*OBX model interaction indicated significantly decreased active responding in the OBX rats on FR5(2), FR5(3), FR5(4) and FR5(5) (FR5(2): *t*_(57,382)_ = −5.67, *p* < 0.001; FR5(3): *t*_(57,382)_ = −5.49, *p* < 0.001; FR5(4): *t*_(57,382)_ = −4.63, *p* < 0.001; FR5(5): *t*_(57,382)_ = −6.38, *p* < 0.001) protocols. Inactive lever pressing did not differ among the groups.

Mean number of delivered food pellets (Figure 5C) showed a significant effect of OBX model (*F*_(1,10)_ = 27.98, *p* < 0.001), schedule of reinforcement (*F*_(5,50)_ = 90.75, *p* < 0.001) and schedule of reinforcement*OBX model interaction (*F*_(5,50)_ = 2.89, *p* = 0.023). Bonferroni *post hoc* test for schedule of reinforcement*OBX model interaction indicated significantly decreased food pellets intake in the OBX rats in the FR5 (*t*_(57,476)_ = −4.41, *p* = 0.003) and FR5(2) (*t*_(57,476)_ = −4.12, *p* = 0.008). All variables are shown in Figure 5 together with an overview of statistical results (right).

#### Evaluation of Sex Differences

For the analysis of sex differences all groups were analyzed together. The variables included in the analysis were active lever presses and cumulative food pellets intake because responding on the inactive operandum did not differ among the groups.

**Figure 6:**
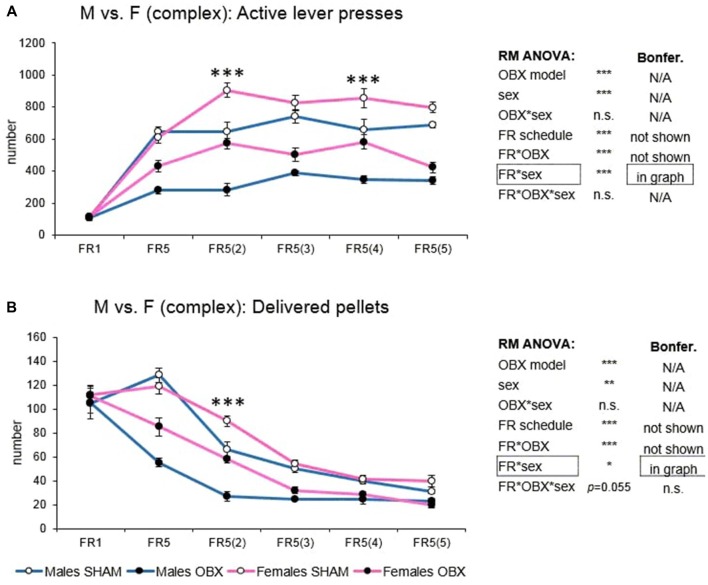
Sex differences in the self-administration of palatable pellets under complex FR5_(x)_ schedule. The graphs present the mean ± SEM of mean numbers of active lever presses **(A)** and numbers of self-administered pellets **(B)** in SHAM and OBX rats of both sexes during each schedule of the complex protocol. The tables on the side summarize results of RM ANOVA and Bonferroni *post hoc* test for each variable (*p < 0.05, **p < 0.01, ***p < 0.001).

In active lever pressing (Figure 6A) RM ANOVA revealed a highly significant effect of OBX model (*F*_(1,20)_ = 172.97, *p* < 0.001), sex (*F*_(1,20)_ = 37.35, *p* < 0.001), schedule of reinforcement (*F*_(5,100)_ = 107.75, *p* < 0.001), schedule of reinforcement*OBX model interaction (*F*_(5,100)_ = 12.83, *p* < 0.001) and schedule of reinforcement*sex interaction (*F*_(5,100)_ = 7.02, *p* < 0.001). Bonferroni *post hoc* test for schedule of reinforcement*OBX model interaction indicated significantly decreased active responding in the OBX rats on all FR5_(x)_ protocols (FR5: *t*_(113,31)_ = −6.78, *p* < 0.001; FR5(2): *t*_(113,31)_ = −8.69, *p* < 0.001; FR5(3): *t*_(113,31)_ = −8.42, *p* < 0.001; FR5(4): *t*_(113,31)_ = −7.17, *p* < 0.001; FR5(5): *t*_(113,31)_ = −8.96, *p* < 0.001). Bonferroni *post hoc* test for schedule of reinforcement*sex interaction indicated significantly higher active responding in female rats on FR5(2) (*t*_(113,31)_ = −6.82, *p* < 0.001) and FR5(4) (*t*_(113,31)_ = −5.36, *p* < 0.001) protocols.

In the mean number of delivered food pellets (Figure 6B) RM ANOVA detected a significant effect of OBX model (*F*_(1,20)_ = 76.35, *p* < 0.001), sex (*F*_(1,20)_ = 11.92, *p* = 0.003), schedule of reinforcement (*F*_(5,100)_ = 142.03, *p* < 0.001), schedule of reinforcement*OBX model interaction (*F*_(5,100)_ = 11.00, *p* < 0.001) and schedule of reinforcement*sex interaction (*F*_(5,100)_ = 2.64, *p* = 0.028). Bonferroni *post hoc* test for schedule of reinforcement*OBX model interaction indicated significantly decreased food pellet intake in the OBX rats in the FR5 (*t*_(118, 26)_ = −9.16, *p* < 0.001), FR5(2) (*t*_(118, 26)_ = −6.06, *p* < 0.001) and FR5(3) (*t*_(118, 26)_ = −4.08, *p* = 0.005) protocols. Bonferroni *post hoc* test for schedule of reinforcement*sex interaction indicated significantly higher food pellet intake in female rats on FR5(2) protocol (*t*_(118, 26)_ = −4.67, *p* < 0.001). All variables are shown in Figure 6 together with an overview of statistical results (right).

## Discussion

This study investigated sex differences in operant responding of OBX animals for food reward and the modulating role played in both sexes by the feeding status (i.e., restricted vs. *ad libitum*) and by the complexity (FR-1 vs. second order schedule) of the reinforcement schedule. Findings showed that: (i) OBX decreases instrumental responding and food pellet consumption significantly but differentially in male and female rats; (ii) sex significantly affects motivation for food, i.e., responding during both the appetitive and consummatory phases of the second order schedule; and that (iii) the *ad libitum* diet reduces food self-administration in males and sham females but not in OBX females.

In line with the pioneering study of Kelly and Leonard (1996) that revealed a deficit in food-motivated behavior of bulbectomized rats, in this study OBX rats displayed a significantly reduced intake of palatable food in comparison to SHAM controls, thus confirming that bulbectomy alters brain reward functions (Kelly and Leonard, 1996; Slattery et al., 2007). Hyposensitivity to normally pleasurable stimuli, i.e., anhedonia, is a cardinal symptom of depression, and changes in the rewarding properties of pharmacological (e.g., drugs of abuse) and natural stimuli (e.g., sucrose, sex, social play) following OBX have been reported, although with some inconsistent results. Most of the drug self-administration studies conducted so far in OBX rats have reported an increased responding for the drug with respect to control (SHAM) rats (Holmes et al., 2002; Kucerova et al., 2012; Amchova et al., 2014; Grecksch and Becker, 2015; Babinska and Ruda-Kucerova, 2017), although one study reported similar responding for cocaine in OBX and control groups (Frankowska et al., 2014). Less consistent are the results from studies investigating the effect of removing olfactory bulbs in processing natural reward. Indeed, a long-lasting reduction in sucrose intake has been reported in olfactory bulbectomized rats (Primeaux et al., 2003; Romeas et al., 2009) although other studies found that preference for sucrose was not altered by OBX (Slattery et al., 2007) or that it was altered in male but not in female rats (Stock et al., 2000). It has also been shown that olfactory bulbs removal affected sexual behavior and partner-preference in male rats (Edwards et al., 1990) and caused sexual impairments in male rats which is unlikely due to an impaired production of gonadal secretions (Larsson, 1975). Intriguingly, OBX also affected sexual behavior in female rats, with effects that vary with the age at ablation and the behavioral system investigated (Lumia et al., 1981). Social play behavior in juvenile rats, also referred to as rough-and-tumble play or play fighting, is another highly pleasurable, rewarding activity (Trezza et al., 2010) that is affected by removal of olfactory bulbs (Beatty and Costello, 1983). Our study confirmed a blunted response to the rewarding properties of natural stimulus (palatable food) in bulbectomized rats, both under a continuous schedule of reinforcement, when a minimal effort is required to obtain the reward, and under a more complex schedule of reinforcement, where work required to obtain the food reward is increased. In line with our findings, the olfactory tubercles have been shown to encode natural reinforcers and process motivational information in mice (Fitzgerald et al., 2014; Gadziola and Wesson, 2016) and to be able to modulate the reward brain circuitry by interfering with the ventral tegmental area dopaminergic neurotransmission (Zhang et al., 2017). Since the mesolimbic dopaminergic system regulates behavioral responsiveness to biologically significant stimuli, including food, our finding that removal of olfactory bulbs significantly decreased self-administration of palatable food strengthens the notion of a hypodopaminergic function in mesolimbic brain areas of OBX rats as reported by an *in vivo* microdialysis study (Ruda-Kucerova et al., 2015a).

Major depressive disorder affects women to a greater extent than men, and numerous sex differences have been reported in animal models of depression (reviewed in Dalla et al., 2010; Franceschelli et al., 2014). Surprisingly, prior research on OBX animals has been conducted mostly with male rats, with very few studies comparing the same behavioral response between males and females, which has left poorly unexplored the possibility that the behavioral changes induced by OBX may develop differently in the two sexes. One study, for example, examined defensive behavior in male and female OBX rats and found no sex differences in this behavioral trait (Stock et al., 2001), but when examining sucrose preference, OBX female rats exhibited significantly lower levels of preference than OBX males and corresponding control animals (Stock et al., 2000). Yet, no study investigated so far the existence of sex differences in the responding pattern and motivational drive for natural or pharmacological rewards. Along with a reduced self-administration of palatable food in OBX vs. sham rats under both FR1 and (FR5_(x)_) schedules of reinforcement, the present study reveals for the first time significant sex differences in responding for natural reward in the OBX model of depression. We found an overall higher responding for palatable food in female than in male sham rats regardless of the complexity of the schedule of reinforcement. These findings are in line with animal studies showing enhanced self-administration of palatable food in female than in male rats (Ruda-Kucerova et al., 2015b; Venniro et al., 2017) and with human studies showing that women scored higher on food binging (MacLaren and Best, 2010) and display greater reward-related neuronal response to palatable food than men (Legget et al., 2018). Accordingly, self-administration of stimulant drugs has been consistently reported to occur at a faster rate in female than male rats (Roth et al., 2004) and females typically display enhanced learning in most operant conditioning tasks (Dalla and Shors, 2009).

Under the complex FR5(x) schedule of reinforcement, OBX female rats showed higher responding than OBX males; however, the opposite was observed in OBX rats under a continuous (FR1) schedule of reinforcement, with OBX males showing higher responding for food than OBX females. This latter finding was rather unexpected also in light of the higher responding typically showed by female rats when allowed to self-administer palatable food under the same experimental conditions (Spierling et al., 2018), and suggests that OBX may render males and females differently responsive to natural rewards depending upon the amount of effort required to obtain the reward. Indeed, among the variables that influence the vigor with which instrumental actions (e.g., lever-pressing), are the cost of responding, with high-effort actions (e.g., complex schedules of reinforcement) being accompanied by smaller elevations in dopamine than low-effort actions (Day et al., 2010; Gan et al., 2010). With this in mind, it is possible to hypothesize that changes in the dopaminergic system induced by bulbectomy occurred differently in the two sexes, leading male and female animals to behave differently when performing low/high effort actions. This study did not monitor the behavior of female rats during the different phases of the estrous cycle, thus leaving unexplored the possible contribution of the ovarian hormones on food reward behavior (Richard et al., 2017). However, the operant paradigm lasted for more than two full-length estrous cycles and the individual rats did not show fluctuations attributable to a specific hormonal phase.

A further outcome of our study was that food restriction increased responding in both sham and OBX groups, and in both sexes, in line with previous evidence that natural satiation attenuates activity in reward-related brain regions (Thomas et al., 2015). This finding is also consistent with previous studies showing that satiety tends to decrease the accumbal efflux of dopamine observed both during feeding and during anticipation of feeding (Wilson et al., 1995; Ahn and Phillips, 1999). Yet, within each group (i.e., sham-males, sham-females, OBX-males, OBX-females), the difference in the responding rate and cumulative number of food pellets earned between restricted and satiated animals was minimal in the female OBX group, suggesting that satiety does not interfere with the reward value of the hedonic food in OBX females.

In conclusion, our study demonstrated for the first time the existence of significant sex difference in responding for palatable food in the OBX model of depression, with diet condition and complexity of instrumental responding differently affecting male and female OBX rats. Future studies will investigate whether chronic antidepressant treatment will be able to revert food reward behavior in OBX rats and, if so, if the effects will be different in male and female animals.

## Author Contributions

JR-K has developed the original idea, performed the statistical analysis, prepared the figures and wrote the first draft of the methods and results. MTZ performed the experiments, collected data and cross-checked the manuscript and references. PA introduced the OBX model to the Italian laboratory, cross-checked the manuscript and references. WF contributed to design the experiments, analyzed and interpreted the data and edited the manuscript. LF has developed the original idea, supervised the experiments and wrote the first draft of the introduction and discussion.

## Conflict of Interest Statement

The authors declare that the research was conducted in the absence of any commercial or financial relationships that could be construed as a potential conflict of interest.
